# The Placenta in Arrested Pregnancy

**Published:** 1890-09

**Authors:** 


### THE PLACENTA IN ARRESTED PREGNANCY.

Obstetricians have long admitted that portions of retained placenta may remain alive and develop into placental polypi. The growth of the placenta in ectopic gestation after the death of the fetus has been observed by several authorities. Dr. Chaput, of Paris, has reported in the *Bulletins de la Societe Anatomique de Paris* two cases where the placenta lived after arrest of normal pregnancy. In the first abortion occurred at the second month, and the placenta was retained for two months. In the second case the fetus died at the third month, and was retained, with its placenta, for two months longer. The child and its appendages were removed, with fatal results to the mother. Both patients were multiparæ, and over thirty-five years of age. The tissue of the placentæ was examined under the microscope. In each case it was found that the placenta had neither atrophied nor continued to develop as in normal pregnancy. A singular abnormal development was detected. The villi had decreased greatly in number, and lost their vessels; on the other hand, those which remained were from double to ten times their normal size, and contained numerous cells, some very large, without distinct nuclei. The villi retained an epithelial lining. Dr. Chaput takes care to remind his readers that although science proves that retained placenta does not necessarily die and decompose, the results of retention are not on that account less serious. Both his cases suffered severely, with most of the usual syptoms. His observations, he further insists, do not prove that the placenta can go on developing indefinitely; nor can we feel sure that it will perish by gradual atrophy rather than by more or less sudden decomposition. In all cases a retained placenta ought to be removed.—*British Med. Journal.*

